# Innovation is needed in creating electronic health records for humanitarian crises and displaced populations

**DOI:** 10.3389/fdgth.2022.939168

**Published:** 2022-09-29

**Authors:** Anmol Shrestha, Jude Alawa, Henry Ashworth, Mohammad Yasir Essar

**Affiliations:** ^1^ California University of Science and Medicine, Colton, CA, United States; ^2^ Stanford University School of Medicine, Stanford, CA, United States; ^3^ Department of Emergency Medicine, Alameda Health System, Highland Hospital, Oakland, CA, United States; ^4^ Hikma Health, San Jose, CA, United States; ^5^ Kabul University of Medical Sciences, Kabul, Afghanistan

**Keywords:** EHR, humanitarian crises, displaced populations, refugee, global healh

## Introduction

By the end of 2020, the United Nations High Commissioner for Refugees (UNHCR) recorded over 82.4 million forcibly displaced individuals worldwide (1). This number is only expected to increase in the coming years because of rising rates of conflict, infectious disease outbreaks, and natural disasters. Displaced populations face distinct health threats, including poor nutrition, conflict-related injuries, and infectious illnesses (2). In addition, because humanitarian crises are now occurring in higher-income countries with comparatively healthier populations, displaced migrants are presenting with a high burden of chronic health conditions, such as cancer and diabetes, that require long-term access to healthcare (3). The rapid influx of refugees with a diversity of pressing health needs presents a number of challenges for host countries. This is challenging in low resource settings given that 84% of those displaced reside in low-to-middle income countries with insufficient resources to provide required health services (4).

In attempting to address these challenges, health providers have historically operated without formal systems for maintaining patient health records. This lack of access to medical records hinders opportunities to establish consistent access to quality healthcare and perpetuates poor health outcomes. Previous research investigating the role of electronic health record (EHR) systems in supporting displaced populations demonstrates their potential to improve patient outcomes by tracking disease markers, increasing provider adherence to treatment guidelines, and increasing patient adherence (5). EHRs have been traditionally developed by and for use in high-income countries. However, as the field of global health continues to decolonize, the transfer of EHR technologies to LMICs will have immense potential for use in humanitarian settings. In light of unprecedented levels of displacement worldwide, further innovation is urgently needed to create EHRs that are uniquely suited to support displaced populations in humanitarian settings.

## Current health records

There have been several EHRs developed to serve the needs of people who have been displaced or affected by humanitarian crises in low-income countries. Many of these EHRs have been developed for one hospital or clinic from the ground up, like the United Nations Relief and Works Agency for Palestine Refugees in the Near East's (UNRWA) “E-Health” EHR platform. E-Health was developed by the UNRWA to document non-communicable diseases, maternal and child health outcomes, and general outpatient visits. It has been successfully used to document and track diabetes mellitus and hypertension in the refugee population in Amman. However, like many other EHRs, E-Health is not open sourced or developed with scalability in mind, thereby making it hard for similar organizations to customize and deploy the platform for their individual humanitarian needs.

On the other hand, two EHRs, Hikma Health and Sijilli, have approached development with scaling across multiple clinics and sites in mind (6, 7). Hikma Health, a non-profit organization based in California, developed a modular, multi-lingual and offline-first capable EHR, using insights and feedback from health care workers, refugee patients and administrators of refugee health clinics in Lebanon and Jordan (5). The platform allows clinicians and administrators to tailor the EHR to capture and report their specific needs. Their user-driven design and modularity has ensured ease-of-use for its users, allowing Hikma Health EHR to deploy in a wide array of settings, ranging from primary health clinics for Syrian refugees in Lebanon to rural health posts in Nicaragua. However, the platform still has room for improvement in two main areas. The first is ease-of-access for patients. Their current platform is still difficult for patients to access their own records outside of the clinic where their health records were originally documented. This is especially important considering the refugees' periodic migrations and need to securely access their health records as they move. In addition, the platform still requires software developer assistance to tailor the platform's open-source code, thereby creating additional barriers for clinic administrators and health care workers when customizing the EHR to their clinic's needs. However, their continued success in low resource settings has shown the importance of both offline first capabilities and user design in the successful creation and deployment of EHRs for displaced populations (8).

Sijilli is a Lebanese cloud based mobile EHR system developed based upon the specific needs of its Syrian refugee patient population, in particular the refugees' frequent migrations between camps (9). This constant fluctuation between settlements generated a need for health records that could be shared securely between multiple health sites in settings with inconsistent internet access. Created by the American University of Beirut (AUB) and EPIC Health systems, an US-based EHR company, Sijilli uses user-friendly tablets and computers to generate a password-protected and advanced encrypted standard (AES) PDF document of patients' health records after patients have visited their clinician. The record is then stored in a key-shaped USB drive, which patients can transport with them throughout their migration between camp sites. Their records can later be unlocked at another site using a unique combination key that consists of, amongst other identifying information, each patient's date of birth and initials. The USB drive allows patients and health care workers to securely access medical records regardless of their location or internet access. However, Sijili, like Hikma, has limited customization functionality and requires trained developers to customize the platform. While Hikma and Sijilli's EHR systems are effective tools in caring for refugees, there is still significant room for improvements in the quest to sustainably ensure high quality of care during humanitarian crises.

## Areas for further innovation

There are three key domains where improvements are needed to better serve displaced populations. These domains include EHR system deployment, EHR clinical implementation, and patient ownership. A central solution to all these areas is no-code EHRs, or EHR development software applications that allow the end user to easily create a customized EHR without the assistance of a trained software developer. A no-code EHR approach is critical for several reasons including: (1) it empowers users to develop an EHR specific to their communities' priorities, (2) It de-colonializes who controls the means to produce EHR solutions, (3) It could greatly decrease the cost to operate and run EHRs by removing software engineers, and (4) It could improve EHR uptake by allowing healthcare providers to engage in creation of the system they use.

### EHR system deployment

A customizable approach to EHR system deployment is critical, especially given the variety of clinical contexts in which displaced populations receive care. The Hikma Health EHR attempts to make its EHR customizable by making it free and open-sourced, but there are still barriers for clinical organizations to customize it for their needs. Therefore, new EHR systems should be linked to a user-friendly software development application that allows for clinic administrators to easily adapt EHRs to meet their clinic's needs. This will decolonize EHR development, putting the power and control to customize an EHR into the hands of the user. A linked software development tool should be available not only for the clinical interface, but also the backend of the EHR to ensure simple maintenance.

Additionally, it is paramount that a number of features seen in current EHRs are maintained. These include the mobile and offline first capabilities to ensure that EHRs are operational in any setting. Also, where possible, EHRs should follow the Hikma Health free and open-sourced model to ensure that there are no financial barriers to deployment.

### EHR clinical implementation

One of the largest and most important barriers to successful implementation of EHRs is clinical user engagement. A lack of provider engagement can lead to poor EHR uptake and increased provider burnout (10). Two solutions to improving clinical implementation include the EHR software development tool and community engagement methods. By creating an EHR software development tool, clinical users will be empowered to collectively work together to generate the interface and clinical workflows they desire. By being in control of developing their EHR, providers will feel more ownership, create the best system that works for them, and achieve successful implementation. This process would also allow for EHRs to be created quickly to meet the needs of humanitarian crises or be updated to meet the needs of health threats such as the COVID-19 pandemic.

Current and future developers of EHRs for these settings should understand how the central themes of community engagement could be used to improve deployment of their systems. This includes using trusted key stakeholders to conduct outreach and empowering clinical users to participate in initial EHR design.

### Patient ownership

One of the main problems with current EHR systems is that patients do not retain the rights and connection to their records. As displaced populations are very vulnerable and the safety of their information is paramount, it is also crucial that they retain the control of their medical information. So far Sijilli has created the best model so far by giving patients a USB with their record and a login to a cloud-based site. These are improvements over paper records or EHRs run only on a single clinic server that do not allow patients access. However, the Sijilli record does not allow patients or providers outside of their system to amend or edit any part of a patient's EHR. While this is in place for security reasons, it prevents patients from having an active medical record that truly belongs to them wherever they are. Future EHRs should prioritize patient autonomy and ownership over their record by giving patients access to their records on a cloud-based server and allowing them to add notes or records.

## Discussion

In addition to the unfathomable stress and trauma of forcibly losing one's home, displaced refugees fleeing from humanitarian crises often deal with a multitude of health challenges. They are not only burdened with communicable diseases that come from inhumane living conditions; refugees are also increasingly dealing with chronic diseases and psychiatric illnesses. Their intense and changing burden of disease calls for health records systems that can ensure the necessary continuity of care needed to manage their complex health needs. The forced migration and the difficulty of managing paper-based health records during these relocations also calls for record systems that ensure ease of access to patients' medical records, regardless of the records' origins. An electronic health record, therefore, can be of great potential in addressing these two needs.

So far, the progress made is commendable; however, there's still a long way to go to increase access to EHRs for the needs of the displaced population. The multitude of problems that arise in conflict settings makes it difficult for clinicians and administrators to immediately recognize both the long- and short-term benefits of EHRs; therefore, it is important to engage both community and clinical leaders and include them in both the development and deployment process of the EHR's implementation. Their input will increase buy-in and the long-term sustainability of the EHR within the clinics and hospital systems they will be deployed in. The creation of no-code EHRs that are tailored to each clinical site's unique needs will therefore be incredibly necessary in ensuring community investment. As we have outlined, the no-code EHR development approach will democratize and decolonize EHR solutions by placing the customization power back in the hands of local staff. In addition, refugees that are displaced in other countries should be able to greatly benefit from this EHR service without any barrier. All future EHR applications need to continually consider the patient's security and the necessity for them to control their health information. With this in mind, it is also important that international organizations such as UNHCR have an important role in the implementation of EHR in all countries with refugees.

As international and local organizations look to provide care for displaced populations worldwide, they should consider the critical role EHRs can have in improving health systems and patient care. In order to facilitate their effective implementation, further innovation is needed to expand access to EHRs by creating no-code EHRs that also consider patient access.
